# Three-dimensional forward-scattering imaging flow cytometry system for single-cell analysis

**DOI:** 10.1063/5.0301435

**Published:** 2025-12-10

**Authors:** Minhong Zhou, Jingjing Zhao, Xinyu Chen, Zunming Zhang, Zhaoyu Lai, Ziqi Zhou, Adam de la Zerda, Yu-Hwa Lo

**Affiliations:** 1 Department of Electrical and Computer Engineering, University of California San Diego, La Jolla, California 92093, USA; 2 Institute of Medical Equipment Science and Engineering, Huazhong University of Science and Technology, Wuhan 430074, China; 3 Department of Structural Biology, Stanford University School of Medicine, Stanford University, Stanford, California 94093, USA

## Abstract

Label-free single-cell 3D imaging is essential for accurate phenotyping by minimizing perturbations to native cellular states and facilitating downstream molecular analyses. Among the available modalities, 3D forward-scattering (dark-field) imaging offers the richest subcellular details but faces challenges from strong transmitted-beam interference. We present a high-throughput 3D forward-scattering imaging flow cytometry system employing optical needle-beam illumination, linear micro-mirror arrays for axial scatter detection, and spatiotemporal deconvolution algorithms. Experimental validations with microstructures, hydrogel beads, and HEK-293 cells confirm the method’s capability for robust subcellular resolution at ∼400 cells/s, enabling advanced label-free cellular diagnostics and analysis. This platform enables label-free, high-content cellular analysis and is readily adaptable to integrated cell sorting and AI-driven classification, offering broad potential for biomedical research and diagnostics.

## I. INTRODUCTION

Label-free single-cell imaging has become pivotal for precise cell phenotyping, enabling the correlation of morphological features with underlying functional and molecular characteristics without perturbing native cellular states.^1,2^ Unlike labeled imaging techniques, label-free modalities preserve intrinsic cellular properties, facilitating subsequent multi-omics analyses and clinical diagnostics.^3–5^ Imaging flow cytometry (IFC) uniquely provides high-resolution images of single cells at subcellular detail in a high-throughput manner, thereby bridging traditional cytometric assays and microscopy.^6–9^

However, current IFC systems predominantly deliver two-dimensional (2D) information,^10,11^ limiting their ability to accurately capture spatial complexity. Critical intracellular structures and interactions may be obscured or inaccurately interpreted when projected onto a 2D plane. For instance, distinct scattering centers in three-dimensional (3D) space can appear overlapped in a single-plane projection, leading to erroneous biological conclusions. Conversely, 3D imaging techniques significantly enhance spatial resolution and reveal dynamic intracellular processes critical for cell biology and biomedical research.^12,13^

Recent progress in 3D IFC has centered on fluorescence tomography,^14–17^ powering AI-driven classification, subtyping, and fate prediction. For example, Hua *et al.* achieved multi-color 3D fluorescence imaging at up to 5750 cells/s.^18^ However, these fluorescence images require labels and pre-treatment of samples, and these processes may disturb cells and create bias for phenotypes. Pirone *et al.* presented a stain-free strategy that combines tomographic phase microscopy with computational statistical-inference segmentation (CSSI) to identify nuclei,^19^ but the approach depends on models trained on fluorescence ground truth and operates at low throughput (∼1 to 2 cells/s).

Building on side-scattering tomography and spatiotemporal mapping, our group previously reported a 3D side-scattering imaging flow-cytometry platform^20,21^ and showed that 3D scattering encoded rich information for cell classification, type discovery, and fate prediction.^22^ In this paper, we incorporate the design of micro-mirror array z-microscopy^23^ into a microfluidic flow cytometer platform to realize high-throughput, label-free 3D forward-scattering (FSC) single-cell imaging. Our solution addresses previous limitations by employing three core innovations: (a) needle-shaped laser illumination to reduce background noise; (b) linear micro-mirror arrays coupled with a photomultiplier tube (PMT) array to achieve axial resolution; and (c) a sophisticated spatiotemporal deconvolution algorithm to reconstruct high-contrast volumetric images from time-resolved signals. The system is broadly compatible with widely used microfluidic chips and cartridges. Validated with microfabricated structures, hydrogel cell simulants, and biological cells (HEK-293), our platform achieves robust subcellular resolution at ∼400 cells per second, establishing a foundation for enhanced diagnostic capabilities, integrated image-activated sorting, and AI-driven cellular analysis.

## II. PRINCIPLE AND METHODS

**FIG. 1.**
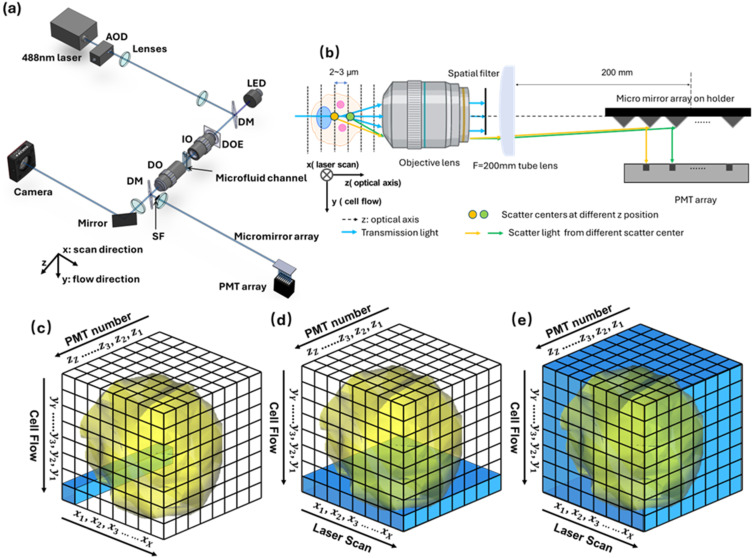
Implementation of the 3D-FSC-IFC system. (a) Schematic diagram of the 3D-FSC-IFC system. AOD: acousto-optic deflector; DOE: diffractive optical element; IO: 20X/0.42 illumination objective lens; DO: 50X/0.55 detection objective lens; SF: spatial filter; BSs: beam splitters; and PMT array: eight-channel photomultiplier tube array. The AOD produces a scanning laser beam, and the DOE generates a needle-shaped beam. The sample is 2D hydrodynamically focused by the sheath flow in a microfluidic cartridge. (b) Illustration of the z-resolution principle: by blocking transmitted light, the spatial filter causes scattered light from different z-positions to focus onto distinct micro-mirror elements and separate PMT channels. (c) 3D reconstructed space. The resolution along the x-axis is determined by the laser scanning range; the resolution along the y-axis is determined by the distance the cell travels between two scans; and the resolution along the z-axis is determined by the number of PMTs and their focal plane spacing. Each time the laser illuminates an object, a 1D intensity profile along the z-axis is recorded. (d) A single laser scanning period produces a 2D xz-plane intensity profile. (e) Once an object fully passes through the scanning area, the time-domain signal contains the complete 3D profile.

Figure 1(a) presents a simplified schematic of the 3D-FSC-IFC system. The 3D-FSC-IFC system employs a 50 mW, 488 nm laser (SF NX 488-50 CDRH, Sapphire). The laser polarization direction is changed from horizontal to vertical by a half-wave plate (WPH10M-488, Thorlabs) before being deflected by an acousto-optic deflector (AOD) (OAD948, ISOMET) driven by a sawtooth voltage signal from a waveform generator (WaveStation 2022, Teledyne LeCroy). This setup creates a scanning laser beam at 200 kHz. A 4f system with two lenses (L1: f = 40 mm, LBF254-040-A, Thorlabs; L2: f = 150 mm, LBF254-150-A, Thorlabs) is used to expand the beam. A 50:50 beam splitter (BS013, Thorlabs) is used to introduce the LED (M455L3, Thorlabs) into the setup to aid visualization and optical alignment. A customized diffractive optical element (DOE) is placed before the input objective (IO) to generate a needle beam. A 20×/0.42 Plan Apo Infinity-Corrected Long WD objective lens (Mitutoyo) was used for illumination. A 50×/0.55 Plan Apo Infinity-Corrected Long WD objective lens (Mitutoyo) is used for detection. The detection objective is mounted on a translation stage to optimize the working distance for different operating conditions. Emergent light is split by a 10:90 beam splitter (BS025, Thorlabs), 10% through lens L3 (LBF254-200-A, Thorlabs) to the camera (CS165CU, Thorlabs) to help focusing and 90% through lens L4 (LBF254-200-A, Thorlabs) to the micro-mirror array. Each micro-mirror in the mirror array is 70 *μ*m high and 5 mm long, fabricated by 3D printing (Nanoscribe Photonics GT) and coated with a 200 nm aluminum layer by sputtering to enhance reflectivity. A 3D printed spatial filter is placed before L4 to block transmission light. Micro-mirror array geometry and objective selection are determined by resolution and fabrication requirements. Full specifications are given in the supplementary material.

The design of the mirror array is described in the supplementary material. A 4f system is used to focus the forward scatter signal from the mirror array into the photomultiplier tube (PMT) array (H9520-20, Hamamatsu). The data acquisition system and the diffractive optical element are also described in the supplementary material.

Cells are delivered via a disposable microfluidic cartridge (NanoCellect) mounted on a translation stage for precise positioning, with flow driven by a fluidic pump system (NanoCellect). Suspended cells are hydrodynamically focused into a single file within the channel. We operate at a mean flow speed of 0.16 m s^−1^, which yields robust one-dimensional hydrodynamic focusing. Under these conditions, the sample volumetric flow is 12 *μ*l min^−1^, and the input cell concentration is limited to ≤2000 cells/*μ*l to prevent aggregation and clogging.

The scanning laser beam at a scanning rate of 200 KHz is a needle-shaped beam formed by a diffractive optical element (DOE), with a diffractive-limited waist of ∼3 *μ*m and a depth of focus of ∼40 *μ*m.^24^ While the laser is rated at 50 mW, the output power for typical operation is ∼3 mW. With a 50:50 beam splitter placed before the sample path, the actual laser power incident on cells is <1.5 mW. At a flow speed of 0.16 m/s, each cell resides in the scanning region for <0.2 ms, corresponding to an exposure of <0.3 *μ*J per cell. This low dose is well below reported photothermal and photochemical thresholds and, therefore, does not perturb cell viability or physiology.^25^

To deconvolute the image signal, we consider that the needle-shaped laser beam maintains a Gaussian profile with a beam width *w*; thus, the time-domain signal recorded by the PMT array can be expressed as

$$\begin{array}{ll} P_{i}\,\left(t\right) & =\sum_{j}\iint_{x,y}dxdyM_{ij}\,\left(y\right)\,\exp\left[\frac{-2y^{2}}{w^{2}}\right]\\ & \;\times f\left(x,\right.v_{y}\left.\,t,z_{j}\right)\exp\left[\frac{-{\left({x-x}_{n}\left(t\right)\right)}^{2}}{2w^{2}}\right], \end{array}$$ (1)

where $P_{i}\left(t\right)$ denotes the signal recorded by the ith PMT in the array and $f\left(x,y,z_{j}\right)$ with $y\left(t\right)=v_{y}t$ represents the scattered light intensity from a traveling object at position $\left(x,y,z_{j}\right)$ at time *t*, in which $z_{j}$ is the focal position in the object space corresponding to the jth PMT. The flow speed of the object is $v_{y}$, while $x_{n}\left(t\right)$ is the laser’s x position at time *t* during scanning, described as

$$x_{n}\left(t\right)=v_{x}\left(t-\frac{T}{2}-nT\right),nT<t<\left(n+1\right)T,n=0,1,2\ldots ,$$ (2)

where $v_{x}$ is the laser scanning speed and T is the scan period ($\left.5\;\mu s\right)$ at a scanning rate of 200 KHz. $M_{ij}\left(y\right)$ denotes the magnification factor relating to the scatter signal from position $\left(x_{n},y,z_{j}\right)$ to the signal recorded by the ith PMT. $M_{ij}\left(y\right)$ does not depend on the x-position because the micromirror is extended through the entire scanning length. For a system with a 50X multiplication factor and a scanning length of 40μm in the object plane, each micromirror in the array has a length of greater than 2 mm. The elements of the matrix, M, depend on the magnification of the system and the design of micromirrors and can be obtained from ray tracing or measurements. $M\left(y\right)$ is a diagonal matrix if there is no crosstalk, meaning that the scattered light from an object at position $z_{i}$ intersects only the ith micro mirror and is detected by the ith PMT. In general, however, the off-diagonal elements in $M\left(y\right)$ are non-zero, and the amount of crosstalk can be obtained from measurements or ray tracing. The detailed process of finding the matrix, M, is elucidated in the supplementary material.

For a scanning frequency of 200 kHz and a scan range of 40 *μ*m, the scanning speed is $v_{x}=8\;m/s$. The flow speed, $v_{y}$, is ∼0.16 m/s. Because the scanning speed is much greater than the flow speed, in our model, for 3D FSC image reconstruction, we assume that the y position of the scattering object remains unchanged during a single laser scan.

The 3D distribution of light scatters, $f\left(x,y,z\right)$, can then be reconstructed by

$$\left[\begin{array}{c} f\left({x,y,z}_{1}\right)\\ \vdots \\ f\left({x,y,z}_{N}\right) \end{array}\right]≅F_{k_{y}}^{-1}\left\{{\left[U_{ij}\left(k_{y}\right)\right]}_{N\times N}^{-1}\left[\begin{array}{c} V_{1}\left(x,\;k_{y}\right)\\ \vdots \\ V_{N}\left(x,\;k_{y}\right) \end{array}\right]\right\},$$ (3)

where

$$U_{ij}\left(k_{y}\right)=F_{y}\left\{M_{ij}\left(y\right)\exp\left[\frac{-2\left(y^{2}\right)}{w^{2}}\right]\right\},$$ (4)

$$V_{i}\left(x,\;k_{y}\right)=F_{y}\left\{F_{k_{x}}^{-1}\left[\exp\left(\frac{\pi^{2}w^{2}{k_{x}}^{2}}{2}\right)F_{x}\left(P_{i}\left(x,\;y\right)\right)\right]\right\},$$ (5)

$${\;P}_{i}\left(t\right)={\;P}_{i}\left(x_{n}\left(t\right),v_{y}t,\;z_{i}\right)≅{\;P}_{i}\left(x_{n}\left(t\right),v_{y}\left(nT\right),z_{i}\right),\;n=0,1,2\ldots .$$ (6)

The above-mentioned equations establish a direct relationship between the time-domain PMT signals and the spatial distribution of scattering centers. The detailed derivations of the analysis, the angular collection property of the scatter signal, and the axial PSF can be found in the supplementary material.

## III. RESULT

### A. Imaging 3D printed structure

**FIG. 2.**
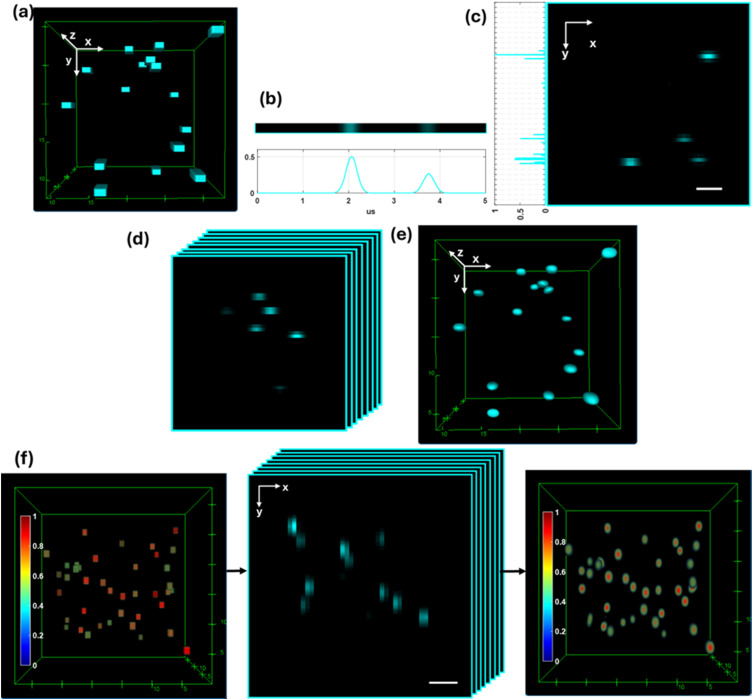
Simulations of 3D FSC image construction from the signals of a PMT array. (a) A simulation sample with two scatter centers at each of the eight focal planes corresponding to the PMT array. The illumination beam propagates along the z-axis and scans in the x-axis at 200 kHz, while the sample flows along the y-axis at 16 cm/s. (b) Signal simulated via Zemax ray tracing. A single laser-beam scanning period produces a 1D light-intensity profile on a single PMT along the x-axis. The PMT’s voltage readout for each sample point corresponds to the light intensity of one voxel in the x-axis. (c) As the object travels along the y-axis, multiple scans generate a 2D profile in the xy-plane by a single PMT signal. (d) Each of the eight PMTs in the array generates a 2D intensity profile at a discrete focal plane along the z-axis. Due to crosstalk, where scattered light from other focal planes can intersect the micromirror, intensities from scattering centers outside the corresponding focal plane can appear in the 2D image. Such a crosstalk is included in the matrix M, and its effect is removed in the 3D image construction using the mathematical formulation in the text. (e) Reconstructed 3D FSC image from the time-domain signals from eight PMTs. (f) A 3D-FSC sample in simulation containing five scatter centers of varying intensities at each PMT’s focal plane, the resulting 2D profiles from the eight PMTs, and the reconstructed 3D profile. Scale bars: 5 *μ*m. Units: μm. The normalized signal intensity is indicated by color.

We first examine the above-mentioned mathematical formulations using simulated signals. We assume that a sample has two scatter centers at the focal plane of each PMT [Fig. 2(a)]. We obtain  $P_{i}\left(t\right)$ and the magnification factor  $M_{ij}\left(y\right)$ of the 3D-FSC-IFC using Zemax ray-tracing simulation. Then, we apply the above-mentioned equations, implemented in MATLAB, to reconstruct a 3D scattering image and compare the reconstructed image with the ground truth. All resulting scatter images are resized to 200 × 200 × 100 pixels for 3D analysis with ImageJ.

The 3D scatter distribution design, simulated signals, and reconstructed 3D FSC images are shown in Fig. 2. The reconstructed 3D FSC image in Fig. 2(e) shows all scatter centers in the sample, having crosstalk from out-of-plane scatters removed. A more complex sample design featuring additional scatter centers of varying intensities is shown in Fig. 2(f), and the reconstructed image accurately reflects the original scatter distribution and its intensities. These results demonstrate that the 3D-FSC-IFC system can reconstruct the 3D-FSC image using the above-mentioned equations.

### B. Imaging 3D printed structure

**FIG. 3.**
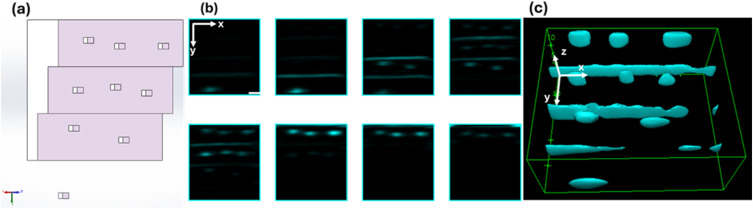
3D-printed sample imaged by the 3D-FSC-IFC system. The illumination beam propagates along the z-axis and scans along the x-axis at 200 kHz with a range of 35 *μ*m. The sample is printed on a glass slide and moves in the y-direction at 1 cm/s. (a) Design of the printed sample “bumps” on each surface. Each terrace has an area of 35 × 8 *μ*m^2^ and a step height of 2.5 *μ*m. Each bump measures 1.5 × 1 × 1 *μ*m^3^. (b) Two-dimensional (2D) FSC image slices in the xy-plane of the printed sample acquired from eight PMTs. Scale bar: 5 *μ*m. (c) Three-dimensional (3D) reconstruction from eight 2D profiles.

We validated the 3D-FSC system against an experimentally derived ground truth by imaging a 3D-printed transparent test structure translating at 1 cm/s. Figure 3(a) illustrates the test structure design. The transparent photoresin sample, comprising an array of rectangular bumps and terraces, was fabricated on a glass slide using a Nanoscribe Photonics GT printer and mounted on an XYZ stage translated along the y-axis via a motorized actuator. Each bump and terrace edge acts as a discrete scattering center. Figure 3(b) presents 2D FSC images captured from individual PMT channels. Bumps on different terraces were captured by different PMTs. Figure 3(c) displays the reconstructed 3D FSC volume obtained from eight PMT channels via micromirror array projection. The reconstructed 3D images match the scatters in the printed structure well, demonstrating that our system can record the spatial distribution of forward-scattered light. Minor discrepancies—missing signals at step edges and center-size variations—are attributed to 3D-printing imperfections.

### C. Imaging porous hydrogel beads as cell simulants

**FIG. 4.**
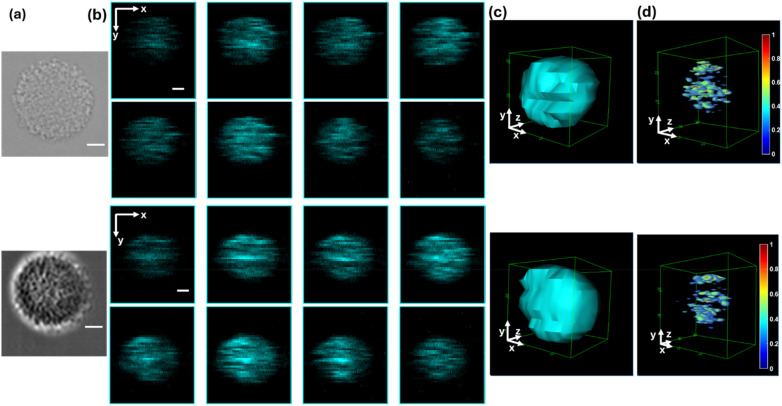
Imaging hydrogel beads by the 3D-FSC-IFC system. The bead diameter is ∼25 *μ*m, and the laser scans a 35 *μ*m range at 200 kHz. The beads flow in a microfluidic cartridge at a speed of 16 cm/s. (a) Representative two-dimensional (2D) bright-field and forward-scattering (FSC, dark-field) images of the hydrogel beads. The bright-field image is acquired via microscopy, whereas the 2D FSC image is obtained using a NanoCellect VERLO image-guided cell sorter. (b) 2D FSC slices in the xy-plane from eight PMTs showing two hydrogel beads. (c) Reconstructed 3D FSC images of two hydrogel beads, shown with a low threshold to highlight the overall volume. (d) Reconstructed 3D FSC images of two hydrogel beads, shown with a high threshold to reveal internal scattering features. Scale bars: 5 *μ*m.

We imaged suspended porous hydrogel beads in the flow (25 *μ*m granular mimic, custom product from Slingshot Bio) as cell simulants to assess the 3D imaging capability of the 3D-FSC-IFC system. Spherical porous hydrogel beads with diameters of ∼25 *μ*m were flowed through a microfluidic channel at a speed of 0.16 m/s. While the 2D bright-field and dark-field images of the beads are shown in Fig. 4(a), Figs. 4(b)–4(d) present the 2D and 3D reconstructed FSC images of the porous hydrogel beads. The reconstructed 3D FSC image with a low threshold is shown in Fig. 4(c), revealing the size and overall shape of the porous hydrogel bead. The reconstructed image with a high threshold in Fig. 4(d) displays more detailed scattering features.

### D. Imaging HEK-293 cells with/without microbeads on the surface

**FIG. 5.**
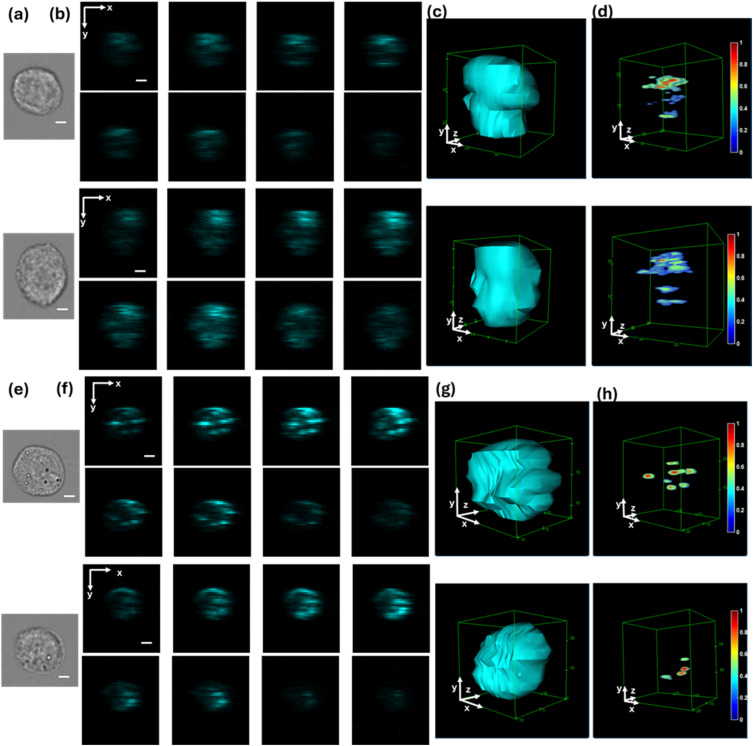
Imaging HEK-293 cells with and without bead attachment using the 3D-FSC-IFC system. The cells flow through a microfluidic cartridge at 16 cm/s, while the laser scans at 200 KHz over a range of 35 *μ*m. (a) Representative microscope bright-field images of HEK-293 cells without bead attachment. (b) 2D FSC image slices in the xy-plane from eight PMTs, showing two HEK-293 cells without attached beads. (c) Reconstructed 3D FSC images of HEK-293 cells, shown with a low threshold to highlight FSC volume. (d) Reconstructed 3D FSC images of bead-free HEK-293 cells, shown with a high threshold to reveal intracellular scattering features. The normalized signal intensity is indicated by the color bar. (e) Representative microscope bright-field images of HEK-293 cells with 1 *μ*m polystyrene beads bonded to the surface. (f) 2D FSC slices in the xy-plane from eight PMTs, showing HEK-293 cells bound with different numbers of 1 *μ*m polystyrene beads. (g) Reconstructed 3D FSC images of HEK-293 cells bound with 1 *μ*m polystyrene beads, shown with a low threshold to highlight FSC volume. (h) High threshold 3D FSC images of HEK-293 cells bound with 1 *μ*m polystyrene beads, demonstrating the number and location of beads attached to the cells. The normalized signal intensity is indicated by the color bar. Scale bars: 5 *μ*m.

To demonstrate the cellular imaging capability of the 3D-FSC-IFC system, we imaged suspended HEK-293 cells at a flow speed of 16 cm/s for a throughput of ∼400 cells/s. Methods for speed compensation and imaging at varying flow speeds are described in the supplementary material. The cells were divided into two groups. One group of cells was bound with a different number of 1 *μ*m fluorescent polystyrene beads, and the other group of cells did not have beads attached. Figure 5(a) shows the microscope bright-field image of a cell without beads, while Fig. 5(e) shows the image of a cell with attached beads. Figures 5(b)–5(d) show the 2D and 3D reconstructed FSC images of the cell without beads. The 3D FSC image in Fig. 5(d), reconstructed by setting a high threshold, shows the cell’s scattering structures. Figures 5(f)–5(h) present the reconstructed FSC images of the cell bound with beads. The high threshold 3D FSC image in Fig. 5(h) reveals the spatial distribution of polystyrene beads attached to the cell. Scatter signals from the beads exceed those from the cells because of refractive index (n) differences among the fluid (PBS, n ≈ 1.335), the cells (n ≈ 1.3–1.6), and the polystyrene beads (n ≈ 1.6).^26,27^ The 3D-FSC-IFC system delineates the FSC volume of cells and indicates bead locations through the high threshold FSC images. Figure 5(h) demonstrates that, although 2D bead images exhibit overlaps, the 3D-FSC-IFC system accurately resolves the precise number of particles from the reconstructed 3D images, thereby mitigating occlusion issues and enabling the detection of both separate and co-localized features.^28,29^

## IV. DISCUSSION

We demonstrated 3D forward-scattering (dark-field) single-cell images in a microfluidic flow cytometer system. The prototype features a throughput of 400 cells/s for a field of view of 40 *μ*m along the x- and y-axes and 20 *μ*m along the z-axis, with spatial resolutions of 1.5, 1.5, and 2.5 *μ*m along the x-, y-, and z-axes, respectively. Increasing the number of micro-mirrors and photomultiplier tubes (PMTs) can enhance resolution along the z-axis. The information-rich 3D FSC (dark-field) images enable label-free assays.^30^ This system offers unique capabilities for biomedical investigations, including early diagnosis and monitoring of malignancies,^31,32^ drug discovery,^33–35^ intracellular structure studies,^36,37^ metabolic engineering,^38^ and cell fate prediction.^39^ The design can be adapted for real-time 3D image-based cell sorting and AI/ML analysis.^40,41^

## SUPPLEMENTARY MATERIAL

The supplementary material includes objective lenses, micro-mirror array, and 4f system; data acquisition; sample preparation; mathematical formulation for the reconstruction of 3D FSC images; angular collection property of the scatter signal and axial PSF; influence of flow speed on cell imaging; and reconstruction method of cell images.

## Data Availability

The data that support the findings of this study are available from the corresponding author upon reasonable request.
